# Supplementary material for: Content-rich biological network constructed by mining PubMed abstracts
Source: BMC Bioinformatics. 2004 Oct 8;5:147. doi: 10.1186/1471-2105-5-147 (PMC528731; doi:10.1186/1471-2105-5-147)
Supplement: Additional File 2 — The original results of the above study (non-essential files are deleted to keep the file size under the limit set by BMC bioinformatics). [file 1471-2105-5-147-S2.bz2 › chilibotAdditionalFile2/dip10/372ID7730326E8295/html/FGF1_FGFR1.html]

 


 **FGF1** and **FGFR1** 
  
Found 826 abstracts in PubMed, retrieved 10.  
 

 What does Google say? 
 PDF only 
| .edu only 

---

**Interactive relationship** (e.g. stimulation, inhibition, etc)

**Non-interactive relationship** (e.g. studied together, co-existance, homology, etc.)

- We show that FGF  [ **FGF1** ]  signaling regulates Pyst1 expression in developing neural plate and limb bud by ablating and or transplanting tissue sources of FGFs and by applying FGF  [ **FGF1** ]  protein or a specific FGFR  [ **FGFR1** ]  inhibitor SU5402.  Ref: 12814546 Curr Biol, 2003
- Analysis of tryptic phosphopeptide maps of FGFR 1  [ **FGFR1** ]  from cells stimulated with FGF  [ **FGF1** ]  2 alone and FGF  [ **FGF1** ]  2 together with heparin showed that FGF  [ **FGF1** ]  2 alone stimulated a several fold increase in tyrosine 463 in the juxtamembrane domain.  Ref: 12799194 Exp Cell Res, 2003
- Fibroblast growth factor FGF  [ **FGF1** ]  FGF  [ **FGF1** ]  receptor FGFR  [ **FGFR1** ]  signaling plays a crucial role in mesoderm formation and patterning.  Ref: 12893744 Circ ResCirc Res, 2003
- We also measured components of the FGF  [ **FGF1** ]  2 ternary signaling complex, fibroblast growth factor receptor 1  [ **FGFR1** ]  FGFR 1  [ **FGFR1** ]  and syndecan 4.  Ref: 12832283 Am J Physiol Lung Cell Mol Physiol< MedlineTA>Am J Physiol Lung Cell Mol Physiol, 2003
- We have studied the mechanism whereby heparin directs FGF  [ **FGF1** ]  2 induced FGF  [ **FGF1** ]  receptor 1 FGFR 1  [ **FGFR1** ]  signal transduction.  Ref: 12799194 Exp Cell Res, 2003
- FGF  [ **FGF1** ]  2 alone stimulated maximal phosphorylation of Src homology domain 2 tyrosine phosphatase SHP 2 and the adaptor molecule Crk, in heparan sulfate deficient Chinese hamster ovary CHO 677 cells expressing FGFR 1  [ **FGFR1** ] .  Ref: 12799194 Exp Cell Res, 2003
- The expression of FGF  [ **FGF1** ]  receptor FGFR  [ **FGFR1** ]  and TGF beta receptor was significantly higher in ALP positive PDLFs than in ALP negative PDLFs.  Ref: 12886987 J Periodontol, 2003
- FGF  [ **FGF1** ]  induced maturation of FGFR  [ **FGFR1** ]  expressing oocytes was blocked by previous injection of Grb7 or Grb14, but not Grb10.  Ref: 12885405 FEBS Lett, 2003
- These changes are associated with specific alterations in the regulation of FGF  [ **FGF1** ]  2, FGFR 1  [ **FGFR1** ]  and syndecan 4 in the airway epithelial mesenchymal trophic unit, which may be associated with the developmental problems of the lung associated with exposure to ozone.  Ref: 12832283 Am J Physiol Lung Cell Mol Physiol< MedlineTA>Am J Physiol Lung Cell Mol Physiol, 2003
